# Epigenetic Dynamics and Regulation of Plant Male Reproduction

**DOI:** 10.3390/ijms231810420

**Published:** 2022-09-09

**Authors:** Quancan Hou, Tianye Zhang, Yuchen Qi, Zhenying Dong, Xiangyuan Wan

**Affiliations:** Zhongzhi International Institute of Agricultural Biosciences, Shunde Graduate School, Research Center of Biology and Agriculture, University of Science and Technology Beijing, Beijing 100024, China

**Keywords:** epigenetics, epigenetic dynamics, epigenetic regulation, plant male reproduction, tapetum, pollen

## Abstract

Flowering plant male germlines develop within anthers and undergo epigenetic reprogramming with dynamic changes in DNA methylation, chromatin modifications, and small RNAs. Profiling the epigenetic status using different technologies has substantially accumulated information on specific types of cells at different stages of male reproduction. Many epigenetically related genes involved in plant gametophyte development have been identified, and the mutation of these genes often leads to male sterility. Here, we review the recent progress on dynamic epigenetic changes during pollen mother cell differentiation, microsporogenesis, microgametogenesis, and tapetal cell development. The reported epigenetic variations between male fertile and sterile lines are summarized. We also summarize the epigenetic regulation-associated male sterility genes and discuss how epigenetic mechanisms in plant male reproduction can be further revealed.

## 1. Introduction

Plants establish a germline cell lineage from somatic cells to complete their sexual reproduction, which differs from animals with a specialized germline cell lineage in early embryogenesis. Male gametophytes are generated and developed in microsporangia differentiated from archesporial cells in flowering plants. Archesporial cells first divide periclinally to generate a primary sporogenous cell inside and a neighboring primary parietal cell outside. Successive divisions of primary sporogenous and primary parietal cells produce meiocytes (the pollen mother cell (PMC)) and three concentric parietal layers, respectively [1]. The concentric parietal layers comprising the anther wall include the endothecium adjacent to the epidermis, the middle layer, and the tapetum that directly contacts the microsporocytes. The PMC undergoes meiosis to generate haploid microspore cells, a process known as microsporogenesis. Haploid microspore cells then undergo microgametogenesis through two rounds of mitotic divisions to form mature pollen (gametophyte). The first mitotic division of each microspore cell is asymmetric and gives rise to a large vegetative cell (VC) and a small generative cell (GC). The GC undergoes a second mitotic division to generate two sperm cells (SCs). Flowering plants such as *Arabidopsis*, rice, and maize disperse tricellular pollen, while many other flowering plants such as tomato and *Medicago* shed bicellular pollen containing one VC and one GC [2]. As the nurse cell, the tapetum plays an essential role in microspore and early pollen development. The tapetum secretes enzymes for releasing PMCs and microspores from the callose wall, and provides nutritive materials for the formation of exine, a pollen wall structure [3]. The processes of pollen mother cell differentiation, microsporogenesis, microgametogenesis, and tapetal cell development are precisely controlled by genetic factors accompanying epigenetic reprogramming, including DNA methylation (especially 5mC, the methylation of the fifth carbon of cytosine) [4], and histone modification changes [5].

Genetic studies have provided a basic view of the regulatory programs underlying plant male gametophyte development. At early anther development, a MADS-box transcription factor (TF) AGAMOUS (AG) [6] activates SPOROCYTELESS (SPL) to promote sporogenous cell specification [7,8]. BARELY ANY MERISTEM 1 and 2 (BAM1 and BAM2) encoding CLAVATA1-related leucine-rich repeat receptor-like kinases (LRR-RLKs) function in promoting somatic cell fates and in restricting SPL expression to the PMCs [9]. Other LRR-RLKs EXCESS MICROSPOROCYTES 1 (EMS1) and SOMATIC EMBRYOGENESIS RECEPTOR-LIKE KINASE1 and 2 (SERK1 and SERK2) form a complex that interacts with TAPETUM DETERMINANT 1 (TPD1) to specify tapetal cell fate [1,10,11,12]. Many tapetum-localized TFs are essential for microgametogenesis, as they regulate the biosynthesis and transport of sporopollenin, which is required for pollen wall formation. A regulatory cascade AtDYT1-AtTDF1-AtAMS-AtMS188-AtMS1 has been well established in *Arabidopsis* [13], and the TF orthologs of the cascade have been proven to play similar roles in rice [14]. In microgametogenesis, key TFs that are involved in asymmetric division, cell cycle progression, and patterning, such as LBD27 controlling pollen mitosis I (PMI) entry and correct asymmetric division [15], MYB81 associated with PMI, and daughter cell lineage formation [16], have been characterized. Readers interested in the genetic regulation of male gametophyte development can refer to available reviews [17,18,19,20].

Epigenetic marks such as DNA methylation and histone modifications are hallmarks that establish the status of chromatins and confer the transcriptional regulation flexibility for plant development and in response to environmental changes [4,21,22]. Over the last decade, plant scientists have investigated epigenetic dynamics and reprogramming during flowering plant reproductive development and have documented dynamic patterns of DNA methylation, histone modifications, and small RNAs in specific plant reproductive cell lineages. The delineated epigenetic states have largely expanded our understanding of the contribution of epigenetic regulation in plant male reproduction. In this review, we summarize the recent progress on dynamic epigenetic changes during pollen mother cell differentiation, meiosis, and microgametogenesis and compare the reported epigenetic reprogramming changes between male fertile and sterile lines. We also summarize the epigenetic regulation-associated male sterility genes and discuss how epigenetic mechanisms in plant male reproduction can be further revealed.

## 2. Epigenetic Dynamics during Pollen Mother Cell Differentiation

PMC specification marks a somatic-to-reproductive cell fate transition and takes place in sporogenous tissues. The transcriptome profiling of *Arabidopsis* male meiocytes revealed that approximately 20,000 genes are expressed, and more than 800 of these genes are preferentially expressed in meiocytes [23]. As expected, these preferentially expressed genes include many genes that encode meiosis-related proteins. Meanwhile, many mitochondrial genes were upregulated in meiocytes compared to anther tissues [24], which may suggest that the meiocytes conduct the energy preparation required for the forthcoming meiosis. Profiling results from two independent research groups showed that the expression of a large number of transposable elements (TEs) was also upregulated in meiocytes compared to other somatic tissues [23,24]. The activation of TEs in meiocytes suggests that the epigenetic states important for TE silencing could be altered.

**Figure 1:**
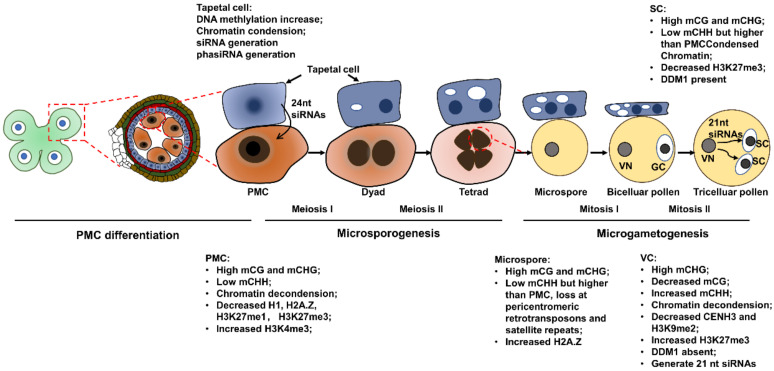
Epigenetic dynamics during plant male reproduction. Epigenetic profiles of each type of cells are described in the text. GC, generative cell; mCG, CG methylation; mCHG, CHG methylation; mCHH, CHH methylation; PMC, pollen mother cell; SC, sperm cell; VN, vegetative cell nucleus.

In *Arabidopsis*, the nuclear morphology of differentiating PMCs was obviously changed, as the nuclei and nucleoli are significantly larger than that of the anther epidermal cells. Meanwhile, the heterochromatin content decreased by 47.9% and the mean number of distinct chromocenters reduced from about 7 to 3 in PMCs. These morphological changes indicate that chromatin decondensation takes place in PMCs. The fluorescence of GFP-tagged linker histone H1.1 and H1.2 was drastically reduced in PMCs, indicating that these H1 variants that are required for chromatin compaction are evicted from PMC chromatin [25]. Similarly, a reduction of DNA methylation and chromatin compaction antagonizing histone variant H2A.Z was also observed in PMCs [26]. The repressive histone marks histone 3 lysine 27 monomethylation (H3K27me1) and H3K27me3 were decreased by 33% and 35% compared to the surrounding somatic cells of the anther wall, respectively. On the contrary, the permissive histone mark H3K4me3 was 1.8-fold higher in the PMC than in the surrounding somatic cells [27]. These chromatin structural alterations and histone modification changes suggest that the PMC establishes permissive chromatin for active transcription. A genome-wide methylation profile of *Arabidopsis* PMCs generated by bisulfite sequencing showed that PMCs feature a typical germline methylome with high CG and CHG methylation levels in TEs but a low CHH methylation level compared to somatic cells (Figure 1) [28]. The decreased CHH methylation could be responsible for the activation of TEs in meiocytes. Domains Rearranged Methyltransferase (DRM) enzymes that establish de novo DNA methylation regardless of sequence context and RNA-dependent RNA polymerase (RDR) are components of the RNA-directed DNA methylation (RdDM) pathway [29]. Most of the sexuallineage-specific DNA hypermethylation in DMRs (differentially methylated regions) were hypomethylated in *drm1drm2* and *rdr2* mutants disrupting the RdDM pathway. Thus, the RdDM pathway is responsible for the DNA hypermethylation in these DMRs. Interestingly, hundreds of sex-lineage-specific DMRs overlapped with genes rather than with TEs, indicating that these DMRs result from RdDM expansion into genic regions. A recent study revealed that the 24-nt small interfering RNAs (siRNAs) present in meiocytes are generated in tapetal cells and mostly target a small number of hypermethylated TE loci in meiocytes, but could also methylate genes with mismatches [30]. This could explain the DRMs observed in genic regions in meiocytes. All 47 differentially expressed genes with an expression change greater than fourfold between *Arabidopsis drm1drm2* and wild type were upregulated in *drm1drm2*. Four of these 47 genes overlapped with sexual-lineage-specific methylated (SLM) loci and were suppressed in meiocytes compared to leaves in wild type. Their expression in leaves was not upregulated by RdDM mutation, indicating that RdDM specifically regulates SLM-associated gene expression in meiocytes [28]. In contrast to the RdDM-directed gene silencing, a meiocyte total small RNA profiling showed that meiocyte-specific small RNAs significantly target genic regions but are positively correlated with gene expression independent of DNA methylation, which is different from mitotic siRNAs that are associated with gene silencing and enriched in intergenic regions [31]. This was the first time RNA-induced epigenetic gene activation (RNAa) was found in *Arabidopsis* and extended our understanding of the two-sided functions of meiocyte small RNAs (sRNAs) in the regulation of meiotic gene expression [31,32].

## 3. Epigenetic Dynamics during Microsporogenesis

**Figure 2:**
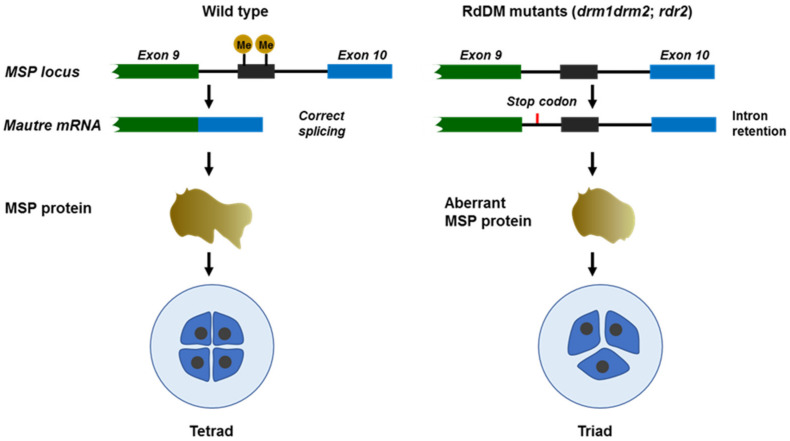
DNA methylation in *MSP* intron is important for correct splicing and normal meiosis.

Microsporogenesis regards the development of microspores (here defined as unicellular microspores) from PMCs, which involve two meiotic divisions [33]. Recently, an allele-specific single-cell RNA sequencing of maize developmental pollen precursors revealed a large gene expression fluctuation during early meiotic prophase I as well as several smaller expression alterations in the following prophase I. The transcriptome from metaphase I to the early unicellular microspore (UM) stage is relatively static [34]. These gene expression shift profiles indicate that the transcription is active during meiosis. A typical event in the first division of meiosis is the recombination between homologous chromosomes. Analyses from different plant species showed that crossovers take place with higher frequencies in gene-enriched chromosome regions than in sub-telomeres with high repeat sequences [35,36,37]. Recombination analysis in *Arabidopsis* and *Mimulus* revealed that crossovers are associated with gene promoter and terminator sites and are strongly influenced by the epigenetic status of the chromatin [38,39]. In *Arabidopsis* and *Mimulus*, recombination signals overlap with the H2A.Z-containing nucleosome, and the crossover frequency is reduced in the *arp6* (*actin-related protein 6*) mutant, in which H2A.Z cannot be deposited to chromatin [38,40]. Although H2A.Z is evicted in PMCs, strong fluorescent signals from GFP-tagged H2A.Z were observed in tetrads, indicating that H2A.Z is reloaded to chromatin during meiosis [27]. Crossovers are also associated with other active chromatin modifications, including H3K4me3 [40]. Similar to PMCs, microspores have high levels of CG and CHG methylation compared to somatic tissues. Although both PMCs and microspores have lower CHH methylation levels than somatic tissues, CHH methylation is partially recovered in microspores (Figure 1) [28]. Pre-tRNA genes encoding phenylalanine and methionine anticodons are hypermethylated in the male sex lineage. Methylation of an SLM-covered methionine pre-tRNA gene located within the last intron of *Multipolar Spindle 1* (*MSP1*) is critical for *MPS1* correct splicing. Loss of the pre-tRNA gene methylation in RdDM mutants *drm1*, *drm2*, and *rdr2* causes intron retention and produces aberrant MPS1 protein, which interferes with the meiosis and leads to triad formation (Figure 2) [28,41].

## 4. Epigenetic Dynamics at Microgametogenesis

Microgametogenesis is the postmeiotic process by which unicellular microspores develop into mature pollens. Mature pollen grains contain three cells resulting from two rounds of mitotic divisions of haploid microspores. Compared to vegetative tissues, the transcriptome of pollen is smaller. A total of 6587 gene transcripts were detected in *Arabidopsis* pollen grains, and these genes are functionally enriched for signaling, vesicle transport, and the cytoskeleton, committing pollen tube germination and tube growth [42].

Immunostaining showed that the active phosphorylated large subunit of RNA polymerase II is distributed throughout the nucleoplasm of VCs in rye pollen but not in GCs and SCs, suggesting that the transcription activity of GCs and SCs is not as active as in VCs [43]. The ability to isolate specific types of cells of male gametophytes has illuminated the transcriptome profiles of SCs and VCs. Gene ontology analyses of *Arabidopsis* and rice SC-expressed genes showed that the ubiquitin-mediated pathway is one of the overrepresented categories [44,45]. In addition, pathways involved in chromatin modeling, small RNAs, and DNA methylation are also enriched, suggesting the epigenetic state is undergoing reprogramming. In *Arabidopsis*, transcript abundances of *AGO9* (Argonaute 9), *DDM1* (Decreased DNA methylation 1), *DRB4* (dsRNA-binding protein 4), *MET1* (DNA methyltransferase 1), and *SUVH5* (SU(VAR)3-9 homolog 5) are so high in SCs that their detection in pollen seems to be SC-derived [45]. Besides *MET1*, several other genes, including *RDR2* (RNA-dependent RNA polymerase 2), *HEN1* (HUA enhancer 1), *DCL1* (Dicer-like 1), and *Pol V* (RNA polymerase V), that are involved in the RdDM pathway are expressed in SCs. However, *DRM2*, *DCL3*, and *Pol IV* (RNA polymerase IV), which are also required for RdDM and *CMT3* (Chromomethylase 3), which function in maintaining CHG methylation, were not detected or were slightly expressed in SCs [45,46].

DAPI (4′,6-diamidino-2-phenylindole) staining of *Arabidopsis* pollen showed that the VN nucleus is more diffuse than SN nuclei, indicating that VN chromatin undergoes decondensation [47]. The VN nucleus chromatin state is correlated with losses of the centromere chromatin epigenetic marks CENH3 and H3K9me2, reminiscent of the nuclei phenotype of the *ddm1* mutant [47]. This is consistent with the high DDM1 expression in SCs but undetectable in VCs [48]. The study in rye pollen revealed that GC and SC nuclei have more H3K9me2 than VC and show a uniform distribution pattern throughout GC and SC nuclei, which is typical for heterochromatin. Immunostaining of H3K4me2 is highly detectable in GC and SC nuclei and distributed towards the nuclear membrane, while the signal in the VC nucleus is weak and dispersed. The acetylation of H3K9 (H3K9ac) was detected in GC and SC nuclei but not in the VC nucleus. On the contrary, H3K27me3 was observed at the telomeric poles of VC nucleus, while a weak signal was detected in GC nuclei and completely undetectable in SC nuclei [43]. A recent study found that H3K27me3 is essential for VC fate commitment. Depleting H3K27me3 in VCs disrupts VC development and leads to a gamete destination, suggesting that H3K27me3 erasure in GC and SC is essential for their fate commitment [49]. While 9 out of 15 *Arabidopsis* histone H3 encoding genes were expressed in somatic cells, only several H3 variants are present in male gametes. Only H3.3 variants HTR5, HTR8, and HTR14 were detected in VCs, and HTR5, HTR10, and CENH3 were expressed in SCs. Since many canonical histones, including CENH3, have been lost in the VC nucleus, this could be one of the reasons for transposon activation in VCs. The HTR10 is male germline-specific, indicating that SCs have a unique chromatin compared with nongametic lineage cell chromatins [50].

Methylome profiling by bisulfite genomic sequencing of individual *Arabidopsis* pollen cell types revealed that VCs and SCs retained high levels of DNA methylation as in PMCs and microspores at CG and CHG sites in the pericentromeric heterochromatin (Figure 1) [28,46]. However, CHH methylation is lost from pericentromeric retrotransposons and satellite repeats in microspores and SCs, but it is restored in VCs by de novo DNA methyltransferase via the RdDM pathway. Although CG methylation was globally retained, the loss of CG methylation at a subset of TEs and intergenic regions was observed in the VCs. Among the identified DMRs, almost all CG DMRs are hypomethylated in VCs, while CHH DMRs are hypomethylated in SCs. Of the 221 VC CG hypomethylated regions, 109 overlapped with different TEs. A quantity of 1781 out of the 2270 CHH DMRs overlapped with different TEs, including 1483 LTR/Gypsy TEs and 139 DNA transposons. These TEs were similarly CHH-hypomethylated in microspores and SCs compared with VCs. The differential methylation profiles of VCs and SCs could be due to the differential expression patterns of DNA methyltransferases and DNA demethylases. The maintenance of CG and CHG methylation in pollen development is consistent with the expression of the DNA methylation maintenance methyltransferases MET1 and CMT3 during microspore and generative cell division [51]. Analysis of the 221 VC CG hypomethylated regions showed that 48 of them are targets of DNA glycosylase DEMETER (DME), and 134 of them are targets of DME homologs ROS1, DEMETER-Like2 (DML2), and DML3. DME is specifically expressed in VCs and the central cell of the female gametophyte, while ROS1, DML2, and DML3 are expressed in somatic tissues and VCs, but all of these DNA demethylases are absent in SCs. This suggests that the loss of CG methylation in VCs results from the activity of DNA demethylases. Similarly, proteins required for CHH methylation, including DRM2, are responsible for the loss of CHH methylation in SCs [46].

Accumulating evidence has shown that the epigenetic state of VCs could influence the transcription and epigenetic profiles of SCs [46,48,52]. For instance, many imprinted genes are flanking with VC CG DMRs. Analysis of 28 imprinted loci, including 12 paternally expressed genes (PEGs) and 16 maternally expressed genes (MEGs), that have a TE within 2 kb of the coding region showed that all 28 TEs lost CG methylation in the VCs. However, only those surrounding MEGs were targeted by 24 nt siRNAs and CHH methylation in SCs. It is speculated that these 24 nt siRNAs are derived from VCs, in which CG methylation is lost and triggers siRNA generation [46]. Although CHH methylation at retrotransposons in VCs is restored, TEs are activated and transpose in VCs and generate 21 nt siRNAs, probably due to the absence of the chromatin remodeler DDM1 in VCs [53]. These VC-derived 21 nt siRNAs are accumulated in SCs and silence the TEs to prevent transposition and ensure transgenerational genome integrity [48].

## 5. Epigenetic Dynamics in Tapetal Cell Development

The tapetum plays an essential role in the formation of microspores and early pollens. It provides nutrients for microspore development, pollen maturation, and enzymes for releasing microspores from their specialized callose walls during the tetrad stage [3,54]. Tapetum development consists of three stages: tapetum specification, tapetum cell binucleation, and cell degeneration through programmed cell death (PCD) [55]. While extensive studies have been performed on profiling epigenetic dynamics of microspores, few have focused on the dynamic epigenetic changes of the nurse cell tapetum. One study analyzed, for the first time, the dynamics of DNA methylation during the tapetum PCD process of *Brassica napus* and *Nicotiana tabacum* using high-performance capillary electrophoresis (HPCE) and the immunofluorescence of 5mC methods [56]. The results showed that the degree of global DNA methylation increased with the development and degeneration of tapetum, reflected by the 5mdC immunofluorescence signal. Meanwhile, results show that tapetum PCD progress is accompanied by Cyt c (Cytochrome C) release, nuclear architecture reorganization, and chromatin condensation. Consistent with the epigenetic changes, the expression of MET and the activity of a caspase 3-like protease that is critical for PCD were increased during PCD [56].

sRNAs include siRNAs and microRNAs (miRNAs), which affect DNA methylation through the RdDM mechanism or post-transcriptionally regulation of gene expression, respectively [57,58]. Phased siRNAs (phasiRNAs) are plant-specific, which are preferentially produced in male reproductive organs in rice and synthesized through the miR2118- and miR2275-dependent pathway [59]. The biosynthesis process of proteins associated with tapetum development affects the content of phasiRNAs, which subsequently affects meiosis [54]. The pattern of the regulation of phasiRNAs is different in plants; 21 nt phasiRNAs are associated with premeiotic events in maize [60] but with meiosis in rice [61]. Expression of 24 nt phasiRNAs is highest at meiosis I in maize and peaks at the end of meiosis in rice. Studies show that 21 nt and 24 nt phasiRNAs contribute to the CHH DNA methylation of the meiocyte genome in maize [62,63]. In rice, 24 nt phasiRNAs dependent on a tapetum-localized bHLH transcription factor bind a meiocyte-specific AGO protein MEL1 [64,65,66]. These findings suggest that tapetum-generated phasiRNAs could be transported to meiocytes. Additional to the 24 nt phasiRNAs, 24 nt siRNAs generated in the tapetum from the transposon loci through the activity of a tapetum-specific chromatin remodeler CLASSY3 (CLSY3) could methylate genes with mismatches in meiocytes in *Arabidopsis*. These tapetum-derived siRNAs also methylate germline transposons, safeguarding genome integrity. Thus, tapetum-derived siRNAs determine the paternal methylome and drive functional methylation reprogramming throughout the male germline [30].

## 6. Epigenetic Variations between Male Fertile and Sterile Lines

To investigate the regulatory roles of DNA methylation in male fertility, studies comparing the DNA methylation levels between male sterile and fertile lines have been performed. DNA methylome analysis between kenaf (*Hibiscus cannabinus*) cytoplasmic male sterility line (P3A) and its maintainer line (P3B) revealed 650 differentially methylated genes (DMGs), of which 313 and 337 are up- and down-methylated, respectively. Combining the transcriptome analysis of the differentially expressed genes (DEGs) between P3A and P3B, 45 genes were identified as both DEGs and DMGs, indicating that these DEGs could be regulated by related DMGs [67]. A similar study conducted in the soybean CMS line NJCMS5A and its maintainer NJCMS5B revealed 3527 DMRs and 485 DMGs. Twenty-five of these DMRs were further validated through bisulfite treatment, and 9 of them were confirmed to have a relationship with gene expression and DNA methylation [68]. A comparison of DNA methylation profiles between the rapeseed (*Brassica napus*) genic male sterile line 7365A and its near-isogenic fertile line 7365B revealed 410 DMGs. Eleven of these DMGs were found to be involved in anther and pollen development, including the male sterility causal gene *Bnams4*, which is deleterious to tapetal plastids. *Bnams4* was hypomethylated in 7365A, and its expression was upregulated [69].

Rice photoperiod-thermo-sensitive genic male sterile (PTGMS) lines can transform from sterility to fertility under short-day and low-temperature conditions. Several studies also investigated the DNA methylation changes in PTGMS lines under different environmental conditions. The investigation of DNA methylation changes in young panicles of rice PTGMS line PA64S using methylated DNA immunoprecipitation sequencing (MeDIP-seq) identified 1258 DMRs between PA64S (sterility) and PA64S (fertility), and PA64S (sterility) was hypermethylated compared to PA64S (fertility). *OsBIM2*, homologous to *Arabidopsis BIM1* that is involved in the brassinosteroid (BR) signaling pathway, was hypermethylated, and its downstream genes were downregulated in PA64S (sterility) [70]. The genome-wide disruption of DNA methylation, especially CHH methylation, was detected in a cotton high-temperature (HT) sensitive line under the HT condition. These DNA methylation changes are associated with 24 nt siRNA levels. The suppression of DNA methylation in the HT-sensitive line leads to male sterility under normal conditions, indicating that DNA methylation-regulated gene expression is responsible for the sterility phenotype. Transcriptome analysis revealed that the expression of sugar and reactive oxygen species (ROS) metabolic pathway-related genes are significantly modulated in anthers under HT due to DNA methylation disruption [71]. These DNA methylation variations between male fertile and sterile lines could result from genetic mutations but could also be the reason for the sterility phenotype of PTGMS lines.

## 7. Epigenetic Regulation-Related Genes That Are Involved in Pollen Development

So far, more than 20 genes related to epigenetic regulation of plant male reproduction have been identified from different plant species (Table 1). The mutation or ecotopic expression of these genes leads to plant male sterility, indicating the importance of epigenetic regulation in pollen development. AtASHR3 (ASH1-related protein 3), also named AtSDG4 (Set Domain Group 4), encodes a histone methyltransferase that contains both PHD and SET domains. AtASHR3 is involved in histone H3 methylation in the inflorescence and pollen grains. Mutation of *atash3* decreases active histone marks H3K4me2 and H3K36me3 in the VC nucleus and suppresses pollen tube growth [72]. AtASHR3 is expressed in anther and interacts with tapetal-localized TF Aborted Microspores (AMSs). The overexpression of AtASHR3 leads to degenerated anthers and male sterility, suggesting AtASHR3 and AMS together could regulate genes involved in pollen development [73]. Another SET domain-containing protein SDG2 mediates H3K4me3 deposition and promotes chromatin decondensation in the pollen VC nucleus during *Arabidopsis* male gametogenesis. The loss of *sdg2* prevents the mitotic division of pollen GCs and impacts pollen germination and pollen tube elongation, indicating that SDG2 functions during male gametogenesis and that SDG2-mediated H3K4me3 is essential for shaping the gametophyte chromatin landscape [74]. Histone acetylation is a type of epigenetic modification that is involved in transcriptional activation and is associated with many developmental processes in eukaryotes [75]. The loss of function of genes involved in histone acetylation alteration and chromatin-remodeling complexes often causes severe developmental defects, even embryo lethality in higher plants [76]. Mutations of *Arabidopsis* ADA2b and GCN5 encode components of histone acetyltransferase complexes, resulting in pleiotropic effects on plant growth and development, including short stamens in flowers and reduced fertility [77,78]. Histone acetyltransferases are highly conserved in eukaryotic plants. A study in *Physcomitrella patens* revealed that PpHAG1 (orthologous to *Arabidopsis* GCN5) controls the maturation of gametangia and that PpSWI3A/B (orthologous to *Arabidopsis* SWI3 subunits, a component of Switch/Sucrose Nonfermenting chromatin-remodeling complexes) is involved in the maturation of the spermatozoids. The mutation of either PpHAG1 or PpSWI3A/B arrests male gametangia development without fertile spermatozoids generation [79]. OsHUB1 and OsHUB2 encode E3 ligases and function on H2B ubiquitinoylation. *oshub* mutants have H2Bub1 loss and reduced H3K4me2 levels, which lead to an abnormal tapetum and pollen abortion [80]. In addition, the centromere-specific histone AtCENH3 controls centromere division during meiosis, which affects both male and female meiosis [81].

The protein complex ASI1 (Antisilencing 1)–AIPP1 (ASI1-Immunoprecipitated Protein 1)–EDM2 (Enhanced Downy Mildew 2) recognizes intragenic heterochromatin and regulates alternative polyadenylation in *Arabidopsis* [92]. The loss of rice OsASI1 impacts the poly(A) site usage of a large number of genes, particularly heterochromatic element-marked genes, leading to severe developmental defects in pollen [82]. OsPEM1 (Pollen Expressed MBD like 1), a methyl-CpG-binding domain (MBD) family protein, members of which have been identified as important DNA methylation “readers” in animals, is a new master regulator of pollen exine development. *pem1* mutants displayed abnormal Ubisch bodies and exines, but its regulatory mechanism is not yet clear [83]. These findings indicate that epigenetic mark reader proteins play essential roles in plant male gametophyte development.

Mutations in most RdDM genes do not cause remarkable developmental defects in *Arabidopsis*. For example, DNA-dependent RNA polymerase IV (Pol IV) is required for epigenetic activation of siRNA accumulation in the pollen grain, but the loss-of-function mutation of *pol iv* does not cause significant pollen defects in *Arabidopsis* [93]. However, knockdown of *CrNRPD1*, encoding the largest subunit of Pol IV, in *Capsella rubella*, resulted in the arrest of postmeiotic pollen development at the microspore stage [84]. An explanation for the essential role of Pol IV in *Capsella* pollen development could be that TEs are distributed much closer to genes in the *Capsella* genome than in *Arabidopsis*. The arrest of *CrNRPD1* microspores was accompanied by the deregulation of genes targeted by Pol IV-dependent siRNAs. A recent study in rice found that an RNA-dependent RNA polymerase encoding gene *OsRDR2* is essential for rice sexual reproduction. The mutation of *osrdr2* abolishes the accumulation of 24 nt siRNAs and reduces global CHH methylation in reproductive organs, leading to male sterility [85]. These studies in different plant species suggest that RdDM activity is selectively essential for some flowering plants, perhaps because of the genome architecture differences.

ARGONAUTE family proteins (AGOs) consist of a variable N-terminal domain and conserved C-terminal, including PAZ and PIWI domains [94]. AGOs can form RNA-induced silencing complexes (RISC) by binding 21–22 nt siRNAs and then mediating RNA cleavage leading to translational inhibition or participating in the RdDM pathway by binding 24 nt siRNAs [95]. In *Arabidopsis*, AtAGO1 is located in the pollen VC nucleus [96], and AtAGO5 accumulates in the SC cytoplasm [97], which makes the major AGO protein substances active in the mature pollen [86]. Weak mutants of *ago1* and *dcl1* showed pollen developmental defects and led to aborted or shrunken mature pollen grains, while strong *ago1* and *dcl1* mutants failed to develop gametes [98,99], indicating their essential role in gametophyte development. The maize *ZmMS28* encodes an AGO family gene ZmAGO5c. The *ms28* mutant shows premature vacuoles at early meiosis and causes male sterility. Analysis revealed that the content of siRNAs and miRNAs in *ms28* anthers is significantly changed, which may regulate tapetum development by cooperating with the sRNA pathway [87]. In rice, the *OsMEL1* gene encodes an AGO protein that plays an important role in the cell division of premeiotic mitosis and ensures the faithful progression of meiosis [64]. Another rice AGO protein OsAGO2 is related to tapetum PCD; it directly regulates the expression of *OsHXK1* through DNA methylation to control the appropriate production of ROS for the proper timing of PCD [88]. Several other sRNAs have also been found to regulate male reproductive development directly. *OsmiR528* can directly target *OsUCL23* to regulate the generation of flavonoids during pollen development and affect the formation of pollen intine [89]. The mutation of *osmir2118* causes severe male sterility in rice. Further analysis reveals that OsmiR2118-dependent 21 nt phasiRNAs in the anther wall are U-rich, and they are distinct from the phasiRNAs in germ cells, demonstrating the functions of miR2118/U-rich phasiRNA in anther wall development [100]. The *Arabidopsis* miRNA *miR159* represses downstream transcription factors *MYB33* and *MYB65* to regulate male reproductive development [90]. Wheat *miR2275* regulates CCR4-associated factor 1 (CAF1), contributes to meiosis, and is associated with the male sterility of a wheat PTGMS line [91].

## 8. Conclusions and Perspectives

Plant male reproduction is a highly dynamic process involving alterations at both the transcriptome and epigenome levels. Understanding the epigenetic dynamics and epigenetic regulation of plant male reproduction will contribute to creating male sterility lines and HT-tolerant male fertility cultivars using epigenetic techniques. Many epigenetic regulation-related genes involved in plant germline development have been identified. Epigenetic reprogramming plays a fundamental role in facilitating the transcription of genes for plant male gametophyte development and configuring SCs with a chromatin state that not only maintains the genome’s integrity but also permits essential gene expression for the next generation. The cell-type-specific profiling of the transcriptome and epigenome has expanded our understanding of the transcriptomic and epigenetic dynamics during flowering plant reproductive development. A recent study on tepal cell-derived siRNAs revealed that the tapetal nurse cell, previously believed to be mainly for nutrient and enzyme supply, has a new role in shaping sperm cell DNA methylation and silencing germline transposons. The activation of TEs in VCs that do not contribute genetic material to the next generation serves as a means to generate respective siRNAs for TE silencing in SCs and ensure transgenerational genome integration. These findings have extended our understanding of epigenetic regulation in plant male reproduction, but most studies have focused on the common DNA methylation 5mC; no or few studies investigate the dynamic changes of other DNA modifications, such as 5-hydroxymethylcytosine (5hmC) [101] and N6-methyladenine (6mA) [102], during plant male gametophyte development. In addition, further investigation is required to link genes associated with the epigenetic regulation of plant male reproduction with the epigenetic status of different cell types of the plant male organ. The single-cell technology [103,104] developed recently and CUT&Tag-based chromatin profiling technology [105] will provide additional and more precise transcriptional and epigenetic dynamic information at the single cell level, which will facilitate the disclosure of mysteries of the epigenetic regulation mechanisms in plant male reproduction.

## Figures and Tables

**Table 1:** Identified epigenetic regulation-related genes that are involved in pollen development.

Gene	Gene Product	Function	Species	References
*AtASHR3(AtSDG4)*	SET-domain protein histone methyltransferase	Histone modification	*Arabidopsis thaliana*	[72,73]
*AtSDG2*	Histone methyltransferases SET DOMAIN GROUP2	Histone modification	*Arabidopsis thaliana*	[74]
*AtSWI3B/3C/3D*	Component of SWITCH/SUCROSE NONFERMENTING (SWI/SNF) chromatin-remodeling complexes	Chromatin remodeling	*Arabidopsis thaliana*	[76]
*AtADA2b*	Histone acetyltransferase	Chromatin remodeling	*Arabidopsis thaliana*	[78]
*AtGCN5*	Histone acetyltransferase	Chromatin remodeling	*Arabidopsis thaliana*	[78]
*PpHAG1*	Histone acetyltransferase of the GNAT family 1	Histone modification	*Physcomitrella patens*	[79]
*PpSWI3A/B*	Component of SWITCH/SUCROSE NONFERMENTING (SWI/SNF) chromatin-remodeling complexes	Chromatin remodeling	*Physcomitrella patens*	[79]
*OsHUB1/2*	Histone monoubiquitination 1/2	Histone modification	Rice	[80]
*AtCENH3*	Histone variant	Chromatin remodeling	*Arabidopsis thaliana*	[81]
*OsASI1*	Antisilencing 1, intragenic heterochromatin reader	Alternative polyadenylation regulation	Rice	[82]
*OsPEM1*	methyl-CpG-binding domain containing protein	DNA methylation reader	Rice	[83]
*CrNRPD1*	The largest subunit of Polymerase IV	RdDM	*Capsella rubella*	[84]
*OsRDR2*	RNA-dependent RNA polymerase 2	RdDM	Rice	[85]
*AtDCL1*	Dicer-like protein	miRNA generation	*Arabidopsis thaliana*	[86]
*AtAGO1/5*	Argonaute	Associate with miRNA	*Arabidopsis thaliana*	[86]
*ZmAGO5c*	Argonaute	Associate with siRNA	Maize	[87]
*OsMEL1*	Argonaute	Associate with siRNA	Rice	[64]
*OsAGO2*	Argonaute	Associate with siRNA	Rice	[88]
*OsmiR528*	microRNA	Gene silencing	Rice	[89]
*AtmiR159*	microRNA	Gene silencing	*Arabidopsis thaliana*	[90]
*TamiR2275*	microRNA	Gene silencing	Wheat	[91]

## Data Availability

All data are shown in the manuscript.
